# Adherence to the Admission Policy in a Local Old Age Psychiatric Unit in Wrexham: A Clinical Audit

**DOI:** 10.1192/bjo.2022.468

**Published:** 2022-06-20

**Authors:** Tajnin Mitu, Nehad Seddiq, Jian Loo, Mark Perera

**Affiliations:** Betsi Cadwaladr University Health Board, Wrexham, United Kingdom

## Abstract

**Aims:**

Elderly patients are more vulnerable due to the higher prevalence of underlying physical health problems. Hence, it is prudent to have a baseline and regular update on the physical health assessment for all old age mental health inpatients. This paper is aimed to discuss the clinical audit findings on the physical health assessment are done in accordance to the trust policy.

**Methods:**

The clinical audit was conducted in a local elderly inpatient mental health unit over a period of a week. The standard used was based on the local Health Board Policy on acute inpatient admission, which includes an admission clerking with details on physical health need and physical examination should be done within 12 hours of admission, blood investigations and medication chart should be completed within two hours of admission, and an ECG (electrocardiography) should be done at the point of admission.

**Results:**

A total of 21 elderly inpatients admission clerking were analysed. It is noted that over 95 cases admission did not adhere to the prescribed standard. Only 67% of the admission clerking was completed within 12 hours, while only 52% of the admission had physical examination done. Only 24% of the admission completed their blood investigations within two hours and 14% of them had ECG done at the point of admission. Although 90% of medication chart was completed within two hours of admission, there is still room for improvement. Feedback from the junior doctors revealed a multifactorial contribution to the failure of meeting the standards: patient being agitated during admission, lack of communication among different teams, lacking an online documentation system on handover, and the heavy workload on junior doctors on venepuncture and ECG.

**Conclusion:**

The clinical audit has shown a huge area of improvement is needed in terms of the physical health assessment and documentation for elderly inpatient psychiatry unit.

We recommend having a good handover system, training more nurses and HCWs in phlebotomy and ECGs, having ward based doctor cover to improve the adherence for future.

We will be presenting this audit in post grad teaching and junior doctor forum with a plan to conduct a regional audit to compare the adherence on the three different hospital under the same health board.

